# The impact of maternal prenatal and postnatal anxiety on children’s emotional problems: a systematic review

**DOI:** 10.1007/s00787-018-1173-5

**Published:** 2018-06-15

**Authors:** Sarah Rees, Susan Channon, Cerith S. Waters

**Affiliations:** Cardiff University, Cardiff, Wales UK

**Keywords:** Perinatal, Maternal anxiety, Child, Emotion, Development

## Abstract

Maternal mental health problems during pregnancy and the postnatal period are a major public health issue. Despite evidence that symptoms of both depression and anxiety are common during pregnancy and the postpartum, the impact of maternal anxiety on the child has received relatively less attention than the impact of maternal depression. Furthermore, the evidence base for the direct impact of maternal anxiety during pregnancy and the postpartum on children’s emotional outcomes lacks cohesion. The aim of this systematic review is to summarise the empirical evidence regarding the impact of maternal prenatal and postnatal anxiety on children’s emotional outcomes. Overall, both maternal prenatal and postnatal anxiety have a small adverse effect on child emotional outcomes. However, the evidence appears stronger for the negative impact of prenatal anxiety. Several methodological weaknesses make conclusions problematic and replication of findings is required to improve the identification of at-risk parents and children with appropriate opportunities for intervention and prevention.

## Introduction

Perinatal mental health refers to a woman’s mental health during pregnancy and the first year after birth. This includes mental health difficulties existing before and persisting into the pregnancy, as well as mental health problems that develop for the first time, or are greatly exacerbated in the perinatal period. Depression and anxiety are the most common mental health problems during pregnancy, with approximately 12% of women experiencing depression and 13% experiencing anxiety at some point, with many women experiencing both [e.g., 1–6]. Depression and anxiety also affect 15–20% of women in the first year after birth [1, 7]. Despite this, an estimated 40–70% of women in the UK have no access to specialist perinatal mental health services [7].

Perinatal mental health problems are a major public health issue. It is well established that maternal mental health difficulties in pregnancy have been associated with preterm labour, poor infant outcomes, and greater cognitive, behavioural, and interpersonal problems in young children [6, 8]. Perinatal depression, anxiety, and psychosis carry a total long-term cost to society (including health and social care use, productivity losses, infant death, emotional problems, and special educational needs) of an estimated £8.1 billion for each 1-year cohort of births in the UK [7], with 72% of this cost relating to the adverse impacts on the child rather than the mother [7]. Perinatal anxiety (when it exists alone and is not co-morbid with depression) costs an estimated £35,000 per mother–child dyad of which £21,000 relates to the mother and £14,000 to the child [7].

The research literature has predominantly focused on postnatal depression [9], which is associated with reduced maternal sensitivity to the child and adverse offspring cognitive, behavioural, and emotional outcomes, particularly for boys [10–13]. Although there is evidence that symptoms of both depression and anxiety are common prenatally, less attention has been paid to the direct impact of symptoms of anxiety occurring during and after pregnancy [3]. In a systematic review of the impact of postnatal maternal anxiety on child development [8], the outcomes were categorised into three domains: somatic, developmental, and psychological. The strongest evidence for an adverse effect of postnatal maternal anxiety exposure was on offspring somatic and psychological outcomes (in which emotional outcomes were embedded) with the evidence for an effect of postnatal maternal anxiety on child developmental outcomes (developmental milestones and cognitive delay) found to be inconclusive.

Research data indicate that greater than typical elevations in maternal perinatal stress, anxiety and depressive symptoms are associated with a wide range of adverse cognitive, behavioural, and neurophysiological offspring outcomes [14]. A dominant hypothesis is that there are prenatal programming effects for psychopathology, a process known as ‘fetal programming’. However, the evidence base for the direct impact of perinatal maternal anxiety on children’s emotional problems lacks cohesion, often embedding such findings within broader child developmental outcomes [15]. This poses a challenge to those who wish to draw upon research in this area to guide clinical practice and further research developments. Therefore, the aim of this review is to systematically summarise studies which measure the impact of maternal perinatal anxiety on child emotional problems to enable future practitioners and researchers to draw conclusions from the findings.

## Methods

The guidance outlined in the Preferred Reporting Items for Systematic Review and Meta-analysis Protocols [PRISMA-P: 16, 17] was followed.

### Search procedures

Articles published between 1900 through January 31st 2017 were systematically identified through 11 electronic databases (PsycInfo, PsychArticles, Pub Med, Medline, BIOSIS citation index, BIOSIS previews, the Science Citation Index, Embase, SCOPUS, Journal citation reports, and Web of Science). Search terms included a combination of database-specific index terms (anxiety, perinatal period, emotional development, and behaviour problems) and individual terms located in the title or abstract (maternal OR mother*, prenatal OR perinatal OR postnatal OR postpartum OR puerperal, child* OR bab* OR infant*, behaviour* OR behavior*, emotion*). The behaviour search terms were used to ensure the search captured emotional outcomes that are embedded within behavioural measures.

### Inclusion/exclusion criteria

All studies that prospectively examined the impact of maternal anxiety in the prenatal and postnatal period on children’s emotional outcomes were considered for inclusion. The database search was restricted to human research articles, written in English, and published in peer-reviewed journals. Studies that did not assess maternal anxiety during the perinatal period, or where anxiety could not be distinguished from other measures (e.g., ‘psychological distress’; a composite variable combining maternal anxiety and depression) were excluded. Articles that focused on maternal effects and not child outcomes or did not assess child emotional problems were also excluded. The titles and abstracts of all studies identified were screened, with those meeting the inclusion criteria selected for full-text evaluation. Reference lists of identified papers were examined, and the bibliographies of key researchers, defined as the authors of the 14 studies that met the inclusion criteria, were reviewed. Related publications were searched using the investigators name(s)/study names and principle investigators were contacted. The co-morbidity of perinatal anxiety and depression is acknowledged, but given that less attention has been paid to maternal anxiety, the current systematic review concentrates primarily on the direct impact of perinatal anxiety on children’s emotional problems.

There is variability within the developmental literature as to how emotional problems are defined with particular terminology often linked to specific scales. For example, studies which measure emotional outcomes using the Child Behaviour Checklist (CBCL) refer to ‘internalizing’ difficulties which represents a composite score of the emotionally reactive, anxious/depressed, somatic complaints, and withdrawn subscales. Other studies refer to ‘emotional symptoms’ (measured by the Strengths and Difficulties Questionnaire; (SDQ) or ‘social–emotional competence’ (e.g., self-regulation, compliance, and interaction with people), measured using the Ages & Stages Questionnaire–social–emotional; (ASQ: SE). Clinical diagnoses are also used for older children. Therefore, for the purpose of the current review, the term ‘emotional problems’ will be used to encompass all definitions used within the reviewed studies.

**Fig. 1:**
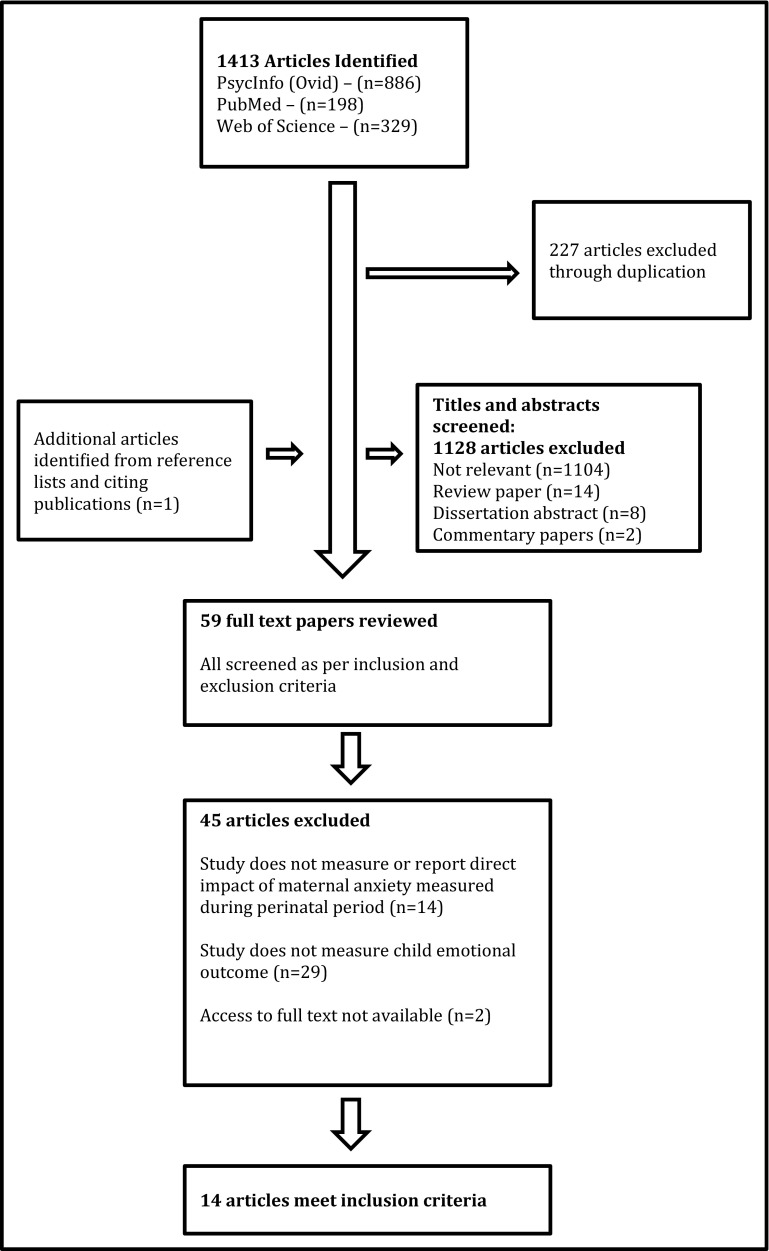
Flow diagram of included and excluded studies

Search results were uploaded to the systematic review software Covidence [18]. Articles from all searches were combined and duplicates removed. A second reviewer independently screened full-text articles and any conflicts were discussed and resolved. A third reviewer was available to discuss screening outcomes in instances where discrepancies and uncertainties between the two reviewers were not resolved. A PRISMA flow diagram reporting the final numbers is shown in Fig. 1.

### Search results

Excluding duplicates, 1,186 studies were identified through database searches. Evaluation of the title and abstracts according to the exclusion criteria decreased the articles from 1186 to 58. Due to prior steps being conservative, several articles that did not meet the review criteria were retained for full-text review, because titles and abstracts were not specific enough to judge for inclusion. Evaluation of the articles’ text reduced the 58 studies to 13. One additional publication was identified through the examination of the reference lists. Fourteen articles were excluded, because studies did not measure or report the direct impact of maternal anxiety measured during the perinatal period. Twenty-nine articles were excluded, because studies did not measure child emotional problems and the full text of two articles could not be accessed. After applying these criteria 14 studies remained, which were published between 2002 and January 2017.

### Assessment of the quality of evidence

The Critical Appraisal Skills Programme (CASP) guidance, recommended for reviewing observational research, was used to aid the appraisal of the included studies. The framework provides an appraisal tool for cohort studies across 12 quality domains with a series of prompt questions for each domain. In each domain, a response of ‘yes’, ‘no’, or ‘can’t tell’ is given. To aid the comparison of quality across studies, a score of two was given for ‘yes’ a score of one was given for ‘can’t tell’ (indicating mixed achievement of the domain) and a score of zero was given to ‘no’. Scores across domains were then summed to give a maximum quality score of 12. The total quality score for each study is included in Table 1 and a summary of the quality scores for each paper reviewed can be found in Table 2.

**Table 1:** CASP quality review for cohort studies framework scores

	Barker et al. [41]	de Brujin et al. [38]	Davis and Sandman [49]	Garthus-Niegel et al. [27]	Leis et al. [42]	Loomans et al. [43]	Murray et al. [40]	O’Connor et al. [46]	O’Connor et al. [48]	O’Donnell et al. [50]	Pickles et al. [45]	Prenoveau et al. [47]	Sharp et al. [39]	Van den Bergh & Marcoen [44]
Did the study address a clearly focused issue?	2	2	2	2	2	2	2	2	2	2	2	2	2	2
Is the population clear?	y	y	y	y	y	y	y	y	y	y	y	y	y	y
Are the factors studied clear?	y	y	y	y	y	y	y	y	y	y	y	y	y	y
Are the outcomes clear?	y	y	y	y	y	y	y	y	y	y	y	y	y	y
Is it clear whether the study tried to detect a beneficial or harmful effect?	y	y	y	y	y	y	y	y	y	y	y	y	y	y
Was the sample recruited in an acceptable way?	1	0	1	0	1	0	0	0	0	1	2	1	2	1
Was the cohort representative of a defined population?	y	n	n	n	n	n	n	n	n	n	y	n	y	n
Was everybody included who should have been included?	n	n	y	n	y	n	n	n	n	y	y	y	y	y
Was the exposure accurately measured to minimise bias?	1	1	1	1	1	1	2	1	1	1	1	1	1	1
Did they use objective measurements?	n	n	n	n	n	n	y	n	n	n	n	n	n	n
Are they valid measures?	y	y	y	Partial	y	y	y	y	y	y	y	y	y	y
Were all the subjects classified into exposure groups using the same procedure?	y	y	y	y	y	y	y	y	y	y	y	y	y	y
Was the outcome accurately measured to minimise bias?	2	1	2	2	2	2	1	2	2	2	2	2	2	1
Did they use objective measurements?	Partial	Partial	n	n	Partial	Partial	y	n	n	n	n	Partial	n	Partial
Are they valid measures?	y	y	y	y	y	y	y	y	y	y	y	y	y	y
Were the measurements methods similar in the different groups?	y	y	y	y	y	y	y	y	y	y	y	y	y	y
Have the authors identified and taken into account of all important confounding factors?	y	y	y	y	y	y	n	y	y	y	y	y	y	n
Was the follow-up of subjects complete enough?	y	partial	y	y	y	y	y	y	y	y	y	y	y	Partial
Was the follow-up of subjects long enough?	y	y	y	y	y	y	y	y	y	y	y	y	y	y
Results	1	1	2	1	1	1	2	2	2	1	1	1	1	1
Were confidence intervals given?	n	n	y	n	n	n	y	y	y	n	n	n	n	n
Are conclusions adequately supported by the results?	y	y	y	y	y	y	y	y	y	y	y	y	y	y
Can the results be applied at population level?	2	0	0	0	2	0	0	0	0	0	2	0	2	0
Total quality score out of 12	9	5	8	6	9	6	7	7	7	7	10	7	10	6

**Table 2:** Methodological characteristics of included studies

Study	Location	Design	Participants and study	Mean maternal age	Measures	Informants on child outcomes	Psychotropic drugs use in pregnancy	Tests of gender differences	Quality rating
Barker et al. [41]	England	Prospective cohort study	3298 mother–child pairs ALSPAC	During pregnancy = 28 years	Mothers: CCEI Prenatal assessments at 32 weeks pregnancy Anxiety not measured in the postnatal period Children: DAWBA aged 7-8 years	Mothers Fathers	Not reported	No	*Q* = 9
de Bruijn et al. [38]	Netherlands	Prospective cohort study	444 women and children	At delivery = 30.7 years	Mothers: STAI (State subscale) and SCL-90 (anxiety scale) Prenatal assessments at 12, 24 and 36 weeks pregnancy Children: CBCL aged 14-54 months	Mothers Fathers	Not reported	Yes	*Q* = 5
Davis and Sandman [49]	Southern California	Prospective cohort study	178 mother–child pairs	At time of assessment = 38.1 years	Mothers: STAI (State subscale) and Pregnancy Related Anxiety Scale Prenatal assessment at 19, 25 and 31 weeks pregnancy Children: CBCL aged 6-9 years	Mothers	Not reported	No	*Q* = 8
Garthus-Niegal et al. [27]	Norway	Prospective cohort study	1472 women and children The Akershus Birth Cohort (ABC)	Mean maternal age not reported	Mothers: Hopkins Symptom Checklist and The Impact of Events Scale Postnatal assessment at 8 weeks postpartum Children: ASQ:SE aged 2 years	Mothers	Not reported	Yes	*Q* = 6
Leis et al. [42]	England	Prospective cohort study	2891 mother–infant pairs ALSPAC	During pregnancy = 29.1 years	Mothers: CCEI Prenatal assessment measured at 18 and 32 weeks pregnancy Postnatal assessment measured at 8 weeks and 8 months postpartum; 21, 33, 61, and 73 months and 11 years of childhood Children: SDQ aged 10–11 years	Mothers Teachers	Not reported	No	*Q* = 7
Loomans et al. [43]	The Netherlands	Prospective cohort study	3446 mothers 3520 teachers 3758 children ABCD longitudinal study	During pregnancy = 31.8 years	Mothers: STAI (State subscale) Prenatal assessment measured at 16 weeks pregnancy Children: SDQ aged 5 years	Mothers Teachers	No –psychotropic drug use excluded from sample	Yes	*Q* = 6
Murray et al. [40]	England	Nested case control study	73 mothers diagnosed with social anxiety disorder and 63 non-anxious controls	Age at follow-up Index group = 36.2 years Control group = 36.6 years	Mothers: SCID; Social Interaction and Anxiety Scale; Social Phobia Scale Prenatal assessment measured at 20–30 weeks pregnancy Children: ADIS-P; CBCL aged 4–5 years	Mothers Teachers	Not reported	No	*Q* = 7
O’Connor et al. [46]	England	Prospective cohort study	7,448 women and children ALSPAC	During pregnancy = 28 years	Mothers: CCEI Prenatal assessment measured at 18 and 32 weeks pregnancy Postnatal assessment measured at 8 weeks and 8 21, 33 months Children: SDQ aged 47 months	Mothers	Not reported	Yes	*Q* = 7
O’Connor et al. [48]	England	Prospective cohort study	6,493 women and children ALSPAC	During pregnancy = 28 years	Mothers: CCEI Prenatal assessment measured at 18 and 32 weeks pregnancy Postnatal assessment measured at 8 weeks, 8, 21 and 33 months Children: SDQ aged 81 months	Mothers	Not reported	Yes	*Q* = 7
O’Donnell et al. [50]	England	Prospective cohort study	7944 women and children ALSPAC	During pregnancy = 28 years	Mothers: CCEI Prenatal assessment measured at 18 and 32 weeks pregnancy Postnatal assessment measured at 33 months. Children: SDQ aged 4, 7, 9, 11 and 13 years	Mothers	Not reported	Yes	*Q* = 7
Pickles et al. [45]	England	Prospective cohort study	813 mothers and infants Wirral Child Health and Development Study	At 20 weeks pregnancy = 26.9 years	Mothers: STAI (State scale); The Pregnancy-Specific Anxiety Scale Prenatal assessment measured at 20 weeks of pregnancy Postnatal assessment measured at 9 weeks, 14 months and 3.5 years Children: CBCL aged 3.5 years	Mothers	Not reported	Yes	*Q* = 10
Prenoveau et al. [47]	England	Prospective cohort study	296 mothers and infants Oxford Parent Project	At 3 months postpartum = 32.3 years	Mothers: GAD-Q; SCID Postnatal assessment measured at 9 weeks and 2, 3, 6, 10, 14 and 24 months. Children: CBCL aged 2 years	Mothers	Not reported	No	*Q* = 7
Sharp et al. [39]	England	Prospective cohort study	316 mothers and infants Wirral Child Health and Development Study	During pregnancy = 26.8 years	Mothers: STAI (State scale) Prenatal assessment measured at 32 weeks of pregnancy Postnatal assessment measured at 5, 9 and 29 weeks, 14 months and 2.5 years. Children: CBCL aged 2.5 years	Mothers	Not reported	Yes	*Q* = 10
Van den Bergh and Marcoen [44]	Belgium	Prospective cohort study	71 mothers and children	During pregnancy between 18 and 30 (mean age not reported)	Mothers: STAI Prenatal assessment measured at 12–22 weeks and 32–40 weeks pregnancy Children: CBCL; STAIC aged 8–9 years	Mothers Teachers	Not reported	No	*Q* = 6

*CCEI* crown crisp experiential index, *STAI* state trait anxiety inventory, *GAD-Q* generalised anxiety disorder questionnaire, *SCID* structured clinical interview for DSM-IV diagnoses, *CBCL* child behaviour checklist, *SDQ* strengths and difficulties questionnaire, *DAWBA* developmental and well-being assessment, *ADIS-P* anxiety disorder interview schedule (parent version), *STAIC* state trait anxiety scale for children

A summary of the effect sizes for significant findings pertaining to the relationship between prenatal and/or postnatal maternal anxiety and children’s emotional outcomes are reported in Table 3. If a study did not report an effect size, but it was possible to calculate an effect size based on the information provided in the published manuscript, post hoc effect sizes were computed for studies reporting significant findings. Only effect size estimates that adjusted for correlated risk factors (e.g., the effect of prenatal anxiety on child emotional problems after controlling for the impact of postnatal anxiety and depression) are reported in Table 3. When it was not possible to extract an effect size, ‘unobtainable’ was recorded in the column. Conventional practice for effect size reporting was followed and small, medium, and large effect sizes were deemed as: Cohen’s *d* = 0.2, 0.5, and 0.8; Cohen’s *f*^ 2^ = 0.02, 0.15, and 0.35; and odds ratios = 1.5, 2.5, and 4.3 [19–21].

**Table 3:** Study analyses, results, and limitations

Study	Covariates	Data analysis	Primary results				Main limitations
			Prenatal anxiety	Effect size	Postnatal anxiety	Effect size	
Barker et al. [41]	Low SES No partner Teen pregnancy Criminal behaviour Substance misuse Cigarette smoking Prenatal depression Postnatal depression Prenatal anxiety Postnatal anxiety	Single path analytic model	Prenatal anxiety predicted increases in child internalizing difficulties after controlling for postnatal anxiety, pre- and postnatal depression and other risk factors	Small	Postnatal anxiety not reported (0 to 12 months postpartum)	N/A	Sample: low rates of ethnic minorities in sample. High attrition rates Measures: did not examine role of prenatal timing of exposure to maternal anxiety or severity of anxiety. All measures based on maternal reports, raises possibility of shared method variance Mechanisms: no measurement of parenting quality as potential mediator or moderator
de Bruijn et al. [38]	Educational level of parents Prenatal smoking Women’s parity Child’s age Postnatal anxiety (mothers and fathers) Postnatal depression (mothers and fathers)	Linear Multivariate Regression	After controlling for confounding factors, significant effects were found for mean prenatal STAI scores on internalizing problems in girls, as reported by fathers No significant effects found for mothers reports	Small	Unobtainable	N/A	Sample: no ethnic diversity (Caucasian only). Participants had higher education level and smoked less during pregnancy Measures: large age range reported on child outcomes Mechanisms: no measurement of parenting quality as potential mediator or moderator
Davis and Sandman [49]	Gestational age at birth Mother’s emotional distress at follow-up Maternal education Child sex	Linear and logistic multivariate regression	Elevated prenatal cortisol and elevated pregnancy-specific anxiety associated with increased anxiety in children following adjustment for covariates. No significant effects of generalised anxiety during pregnancy	Small	Not investigated	N/A	Sample: low rates of ethnic minorities. Low rates of low SES Measures: all measures based on maternal reports, raises possibility of shared method variance Mechanisms: did not account for exposure to extreme stress. No measurement of parenting quality as potential mediator or moderator
Garthus-Niegal et al. [27]	Postnatal depression Postnatal anxiety Age at delivery Maternal education Gestational age Current child health problems Child gender	Linear multivariate regression	Not investigated	N/A	After adjustment for covariates, postnatal PTSD symptoms predicted increased socio-emotional problems, particularly for boys. No effects for symptoms of generalised postnatal anxiety	Small	Sample: selective attrition. Education and symptoms of depression were more likely to drop out. Measures: all data based on mother reports. Relationships could have occurred due to common method bias Mechanisms: No measurement of parenting quality as potential mediator or moderator
Leis et al. [42]	Marital status Maternal age at birth Child birth weight Child gender Maternal educational attainment Cigarette smoking Alcohol use Postnatal Anxiety Pre- and postnatal Depression	Linear multivariate regression	After adjustment for covariates, elevated symptoms of prenatal anxiety predicted increased offspring emotional problems. Finding significant for maternal, but not teacher reports on child outcome	Small	Unobtainable	N/A	Sample: high attrition rates limits confidence in generalizability of results Measures: did not examine role of prenatal timing of exposure to maternal anxiety Mechanisms: did not account for family functioning, paternal mental health. No measurement of parenting quality
Loomans et al. [43]	Birth weight Gestational age Parity Ethnicity Maternal educational level Prenatal smoking Prenatal alcohol use Current anxiety, stress & depression Parental history of psychopathology	Linear multivariate regression	After controlling for covariates, elevated prenatal anxiety symptoms predicted increased child emotional problems, as reported by mothers. No effects on teacher reports after controlling for covariates	Small	Not investigated	N/A	Sample: non-random sample attrition. Women who were younger, less educated, did not have a western background, and were more anxious were less likely to participate Measures: significant findings only for maternal reports, raises the possibility of shared method variance Mechanisms: no measurement of parenting quality as potential mediator or moderator
Murray et al. [40]	Child gender Mother’s IQ Birth order Maternal age SES	Linear or logistic multiple regression	Not investigated	N/A	Children of socially anxious mothers had significantly increased emotional problems as reported by mothers. This effect was moderated by infant attachment style and only significant for securely attached children. No significant findings For teacher reports	Small	Sample: low rates of ethnic minorities and low SES Measures: CBCL and ADIS-P relied on maternal reports—potentially confounding effects of the maternal disorder and shared method variance Mechanisms: Did not examine father or family members who might compensate for difficulties experienced by mothers. Did not control for prenatal anxiety
O’Connor et al. [46]	Gestational age Birth weight Mode of delivery Parity Smoking Alcohol consumption SES Maternal education Maternal age Prenatal and Postnatal depression Postnatal anxiety	Hierarchical logistic regression	After controlling for covariates, prenatal anxiety at 18 weeks predicted elevated emotional problems in girls Prenatal anxiety at 32 weeks gestation predicted elevated emotional problems in boys and girls	Small	Postnatal anxiety at 8 weeks predicted elevated emotional problems in boys and girls. Postnatal anxiety at 8 weeks was also a predictor of emotional problems in girls aged 4 years	Small	Sample: sample attrition was not random. Attrition was more likely in those with higher anxiety scores at earlier assessments. This could result in a diminished effect of prenatal anxiety Measures: all data based on mother reports, therefore, shared method variance could explain the effects Mechanisms: physiological mechanisms not accounted for. No measurement of parenting quality as potential mediator or moderator
O’Connor et al. [48]	Maternal depression Postnatal Anxiety Gestational age Birth weight Mode of delivery Parity Prenatal smoking Prenatal alcohol use SES Maternal education Maternal age	Logistic regression	After controlling for covariates, elevated prenatal anxiety at 32 weeks predicted increased emotional problems in boys but not girls	Small	After controlling for covariates, elevated postnatal anxiety at 8 weeks predicted increased emotional problems in boys but not girls	Small	Sample: attrition was not completely random. Those who dropped out were more likely to be initially anxious and at greater psychological disadvantage Measures: all data based on mother reports, therefore, shared method variance could explain the effects Mechanisms: no measurement of parenting quality as potential mediator or moderator
O’Donnell et al. [50]	Maternal SES Parenting behaviour Maternal age Smoking Alcohol/substance use Birth weight Gestational age Maternal postnatal depression & anxiety Paternal pre- and postnatal anxiety	Longitudinal growth model	Elevated prenatal anxiety predicted persistently higher emotional problems across childhood with no diminishment of effect into adolescence	Small	Unobtainable	N/A	Sample: low rates of ethnic minorities Measures: all data based on mother reports, therefore, shared method variance could explain the effects Mechanisms: did not have direct measures of biological mechanisms that might mediate the effect. No measurement of parenting quality as potential mediator or moderator
Pickles et al. [45]	Maternal age Marital status Education SES Smoking Alcohol consumption Sex of infant Birth weight Postnatal depression and anxiety	Regression	Frequency of infant stroking modified the association between pregnancy-specific anxiety and maternal ratings of offspring emotional problems. The effect of prenatal generalised state anxiety no longer significant following adjustment for confounders	Small	Unobtainable	N/A	Sample: low rates of ethnic minorities Measures: all data based on mother reports. Relationships could have occurred due to common method bias Mechanisms: the data do not allow determination of what physiological mechanisms account for the observed associations
Prenoveau et al. [47]	Mothers age Infant age Infant sex Infant birth order Mother marital status Postnatal depression Maternal anxiety Maternal depression	Latent trait-state occasion modelling	Not investigated	N/A	Maternal anxiety trait or state factors did not predict child emotional problems after controlling for the depression trait factor	Unobtainable	Sample: low rates of ethnic minorities Measures: all data based on mother reports, therefore, shared method variance could explain the effects Mechanisms: did not examine physiological mechanisms that could account for the observed associations
Sharp et al. [39]	Psychological abuse Breast-feeding Mother’s age Marital status Education & SES Prenatal Smoking Alcohol consumption Infant sex Birth weight Maternal sensitivity Postnatal anxiety Postnatal depression	Generalised Linear Latent and Mixed Models	After controlling for covariates, prenatal anxiety predicted elevated child emotional problems for female offspring. High maternal stroking eliminated the association between prenatal anxiety and child emotional problems	Unobtainable	Unobtainable	N/A	Sample: low rates of ethnic minorities Measures: all data based on mother reports, therefore, shared method variance could explain the effects Mechanisms: did not examine physiological mechanisms that could account for the observed associations
Van den Bergh and Marcoen [44]	Parental education Prenatal Smoking Birth weight Child gender Postnatal anxiety	Hierarchical regression	After controlling for covariates, prenatal anxiety at 12–22 weeks gestation was significantly associated with child self-reported anxiety No significant associations for mother or teacher-reported emotional problems	Small	Unobtainable	N/A	Sample: small sample, all Caucasian. Limited generalisability Measures: inconsistent findings across informants and measures Mechanisms: no measurement of parenting quality as potential mediator or moderator

## Results

### Design

Table 1 provides an overview of the included studies. Thirteen of 14 were population-based cohort studies and one used a nested case–control design. Thirteen studies began in pregnancy and one began in the postnatal period. All of the studies used a prospective longitudinal design. Seven studies examined separate study populations, while seven reported different analyses of two birth cohorts (five from one cohort and two from another).

### Participants

All of the studies involved community-based samples of mother–child dyads. Sample sizes ranged from 71 to 7,944 mother–child pairs (median 1143). Ten studies reported mean maternal ages at pregnancy (range 26.8–31.8 years), two reported mean maternal age at assessment and two did not report details of mean maternal age. Children ranged from 14 months to 13 years. Twelve studies assessed children at one timepoint, one study assessed children at two timepoints (4 and 6 years) and one study assessed children at multiple timepoints (4, 7, 9, 11.5, and 13 years).

### Maternal anxiety measures

A number of different measures were used to evaluate maternal anxiety including self-report scales and standardised clinical interviews. The most frequently used measure was the State Trait Anxiety Inventory (STAI) [22, 23], which was used in six studies. The STAI has acceptable reliability and established validity and is one of the most widely used measures of anxiety symptoms [24]. Five studies measured symptoms of anxiety with the 8-item anxiety subscale of the Crown Crisp Experiential Index [CCEI; 25]. The CCEI is a well-validated self-report symptom scale and the anxiety subscale has frequently been used to measure anxiety in perinatal women [e.g., 3, 26]. One study used the Hopkins Symptom Checklist [27]. Three studies used self-report questionnaires that measure specific anxiety disorders and two used the Structured Clinical Interview for DSM-IV Diagnoses [28]. Four studies measured maternal anxiety only in the prenatal period, eight measured maternal anxiety both pre- and postnatally and two measured maternal postnatal anxiety only.

### Child emotional problems and outcome measures

Emotional outcomes were measured using a variety of questionnaire scales and clinical interviews with assessments ranging from 14 months to 13 years. The most frequently used measure, in seven studies, was the Internalizing Scale of the CBCL [29, 30]. Four studies used maternal reports only, two studies included both maternal and paternal ratings, and four studies reported on teachers’ ratings [31]. Five studies used maternal reports of children’s emotional problems on the SDQ [32], an adaptation of a widely used index of psychiatric symptoms in children [33]. One study used maternal reports of children’s social–emotional competence using the ASQ: SE [34]. Another study used both maternal and paternal reports of children’s emotional problems using the Development and Well-Being Assessment, a standardised clinical interview that yields psychiatric diagnoses [DAWBA; 35]. Two studies measured child anxiety specifically alongside the CBCL: one used the Anxiety Disorder Interview Schedule, Parent Version [ADIS-P; 36] and the other used the self-report State Trait Anxiety Scale for Children [STAIC; 37].

### Quality of studies

Table 2 provides an overview of the quality ratings of published articles. The quality of the studies varied from a lowest score of five [38] to a highest of 11 out of 12 [39], with higher scores indicating better quality. Six studies used a second informant (e.g., teacher rating or father rating) in addition to maternal ratings of children’s emotional problems [38, 40–44]. The results of three studies were representative of the general population [39, 41, 45].

## Perinatal anxiety and children’s emotional problems: synthesis of the findings

Table 3 provides an overview of the study findings. Across the identified studies, child emotional problems have been assessed at different developmental stages, with the majority of studies focusing on the early and middle childhood period. Most studies assessed maternal anxiety in the prenatal period with fewer studies assessing anxiety in the postnatal period. Thus, the findings are reported according to stages of childhood and timing of anxiety measurement (Table 3). It is noteworthy that across the 14 studies represented in Table 3, any significant association between pre- and/or postnatal maternal anxiety and offspring emotional problems represents a small effect size following the statistical adjustment of correlated risk factors.

### Early childhood emotional outcomes: the effect of prenatal anxiety

After controlling for covariates, and when mothers reported on child outcomes, 4 studies found prenatal anxiety to be associated with elevated offspring emotional problems when children were aged between 2 and 5 years [39, 40, 43, 46]. Similarly, Pickles et al. found an initial effect of prenatal anxiety on offspring emotional problems at 3.5 years [45]. However, following adjustment for confounding factors (e.g., postnatal anxiety and depression), the association between prenatal anxiety and offspring emotional problems was no longer significant [45]. This particular study had a high-quality rating based on its strong methodology [45].

Three studies used multiple informants (mothers, fathers, and teachers) to assess offspring emotional problems in early childhood [38, 40, 43]. Murray and colleagues found that compared to children of non-anxious mothers, the offspring of socially anxious mothers were themselves more likely to be diagnosed with social anxiety disorder [40]. However, in this study, the clinical diagnosis was based on maternal report and when teacher-rated emotional problems were used as the outcome variable, no significant relationships were observed. This is a surprising finding as one would expect children to show difficulties with social anxiety in the school environment. Thus, this finding may indicate method variance, where elevated associations between maternal anxiety and children’s emotional problems are a result of mothers’ reports being used for both variables. One study found a significant effect of prenatal anxiety on paternal but not maternal reports of female children’s emotional problems [38]. This study scored the lowest quality rating on the CASP and a wide age range was used for the child assessment (14–54 months). Loomans and colleagues [43] found a significant positive association between prenatal anxiety and teacher-rated emotional problems; however, this association was no longer significant after controlling for maternal postnatal emotional distress (composite measure of depression, anxiety, and stress) [43].

### Early childhood emotional outcomes: the effect of postnatal anxiety

In early childhood, the impact of postnatal anxiety on child emotional problems was assessed by three studies. Garthus-Niegel and colleagues [27] found mothers’ Post Traumatic Stress Disorder (PTSD) symptoms at 8 week postpartum predicted elevated offspring socio-emotional problems at 2 years [27]. In contrast to the PTSD symptoms, maternal reported symptoms of generalised anxiety in this study were not significantly related to offspring emotional problems after controlling for confounders [27]. O’Connor and colleagues [46] found postnatal anxiety assessed at 8 week postpartum predicted emotional problems in both boys and girls at aged 4 years [46]. Prenoveau and colleagues [47] found mothers’ persistent postnatal anxiety symptoms measured at 9 weeks and 2, 3, 6, 10, 14, and 24 month postpartum predicted mothers’ reports of children’s emotional problems at 24 months. However, when mothers’ depressive symptoms were included in the model, persistent postnatal anxiety did not independently predict maternal reported emotional problems above and beyond the impact of maternal postnatal depression.

### Middle childhood emotional outcomes: the effect of prenatal anxiety

Three studies assessed how maternal prenatal anxiety impacted on offspring emotional problems in middle childhood (approximately 6–9 years of age). Barker and colleagues [41] found that prenatal anxiety, measured at 32 weeks gestation, was associated with a small increase in children’s emotional problems at 7–8 years of age [41]. Similarly, O’Connor et al. [48] found high levels of prenatal anxiety, measured at 32 week gestation, predicted emotional problems for boys and girls aged 6–7 years after adjustment for the covariates [48]. Similarly, another study found pregnancy-specific anxiety predicted elevated offspring anxiety at aged 6–9 years [49]. However, in this study, prenatal maternal state anxiety was not significantly associated with elevated offspring anxiety in middle childhood [49].

### Middle childhood emotional outcomes: the effect of postnatal anxiety

Only one study examined the impact of maternal anxiety during the first postnatal year on offspring emotional problems in middle childhood. O’Connor and colleagues [48] found that postnatal anxiety at 8 weeks was a significant predictor of children’s emotional problems at 6 years; this result was significant for boys but not for girls [48]. Although postnatal anxiety was significantly associated with emotional problems in boys, it did not eliminate the effect of prenatal anxiety [48].

### Late childhood emotional outcomes: the effect of prenatal and postnatal anxiety

The association between maternal prenatal anxiety and offspring emotional problems in late childhood (approximately 9–12 years of age) was assessed by two studies. Van den Bergh & Marcoen [44] found that prenatal anxiety measured at 12–22 week gestation was a significant predictor of children’s, but not mothers’ or teachers’ reports of emotional problems in late childhood [44]. However, this study did not control for the impact of prenatal or postnatal depression on offspring outcomes. In another study, Leis and colleagues found that elevated symptoms of prenatal anxiety were associated with mother and teacher-reported emotional problems in 10–11-year-old children [42]. Importantly, in this study, the association between exposure to prenatal anxiety symptoms and offspring emotional problems remained significant after controlling for mother-reported prenatal and postnatal depression, postnatal anxiety, and other psychosocial risk factors [42]. The initial significant association between prenatal anxiety exposure and teachers’ reports of children’s emotional problems was not maintained after the influence of the covariates was considered [42]. The reporting of associations between postnatal anxiety and children’s emotional problems was limited in this study [42].

### The effects of prenatal anxiety across childhood

O’Donnell, Glover, Barker & O’Connor [50] measured children’s emotional problems using the SDQ on five occasions from age 4–13 years [50]. The correlations between maternal prenatal anxiety and offspring emotional problems were statistically significant across the 5 timepoints (offspring ages 4, 7, 9, 11.5, and 13 years). The correlation coefficients were almost identical when prenatal anxiety was measured at both 18 and 32 week gestation—suggesting no/minimal timing effects [50]. In addition, longitudinal growth analyses found that child emotional problems changed over time in a U-shaped manner, with lower scores at age 9 years than at 4 or 13 years.

## Maternal prenatal anxiety and children’s emotional problems: mechanisms of effect

Three studies examined potential moderators of the association between maternal prenatal anxiety and child emotional problems. Murray and colleagues [40] found that children of mothers with prenatal social anxiety had higher levels of emotional problems than children of non-anxious controls [40]. When examining whether this relationship was moderated by child attachment style, the effect of maternal social anxiety was significant for securely attached children but not for those who were insecurely attached. Although prenatal anxiety at 20 weeks was found to be predictive of emotional problems at child age 4–5 years, Murray et al. [40] did not control for the effects of prenatal or postnatal depression [40]. Sharp and colleagues [39] hypothesised that maternal stroking would modify the association between prenatal generalised state anxiety and child emotional problems [39]. In this study, with increasing prenatal anxiety, the daughters of low stroking mothers showed increasing emotional problems, whereas this effect was not observed in girls, whose mothers were in the high stroking group, nor was it seen in boys. This study received the highest quality rating. Analyses based on the same cohort by Pickles et al. [45] examined whether the effect of early maternal stroking was still evident 1 year later [45]. After controlling for postnatal anxiety and depression, the frequency of infant stroking modified associations between pregnancy-specific prenatal anxiety, but not generalised prenatal state anxiety, and mothers’ ratings of children’s emotional problems [45]. There was no indication of a sex difference and the test of the three-way interaction; pregnancy-specific anxiety by stroking by sex of child was non-significant. Despite both these studies being derived from the same cohort and having high-quality ratings, different measures of prenatal anxiety were used and the sample size varies considerably which makes the results difficult to compare.

## Discussion

### Summary of findings

This review systematically evaluated the evidence relating prenatal and postnatal maternal anxiety to children’s emotional problems at different phases of development. Based on the findings of the 14 papers which met the inclusion criteria, there was evidence for a small effect of prenatal maternal anxiety [39–44, 46, 48, 50] and pregnancy-specific anxiety [45, 49] on child emotional problems following the adjustment for correlated risk factors. There was also preliminary evidence for the impact of postnatal anxiety on child emotional problems [27, 41, 46–48] again representing a small effect following the adjustment for correlated risk factors. However, the low number and overall quality of the studies including postnatal anxiety render this an unreliable conclusion that requires further research. Across the included studies, several methodological weaknesses limit the ability to draw definitive conclusions.

### Sample characteristics

Half the included papers report findings from two longitudinal cohort studies, five from the Avon Longitudinal Study of Parents and Children study [ALSPAC; 51] and two from the Wirral Child Health and Development Study [39, 45]. Therefore, a combination of limited geographical coverage and study attrition due to missing data and exclusion criteria, mean that the findings might have limited generalisability.

### Maternal anxiety measurement

Assessment of maternal anxiety at different stages of pre- or post-pregnancy makes the studies difficult to compare. All of the studies relied on naturally occurring variations in maternal anxiety in community not clinical populations and the longitudinal designs with large community samples were strengths of most studies. However, selective attrition could mean that studies were examining associations among the less vulnerable individuals. For example, attrition analysis in one study found that mothers who did not provide data at the assessed timepoint were more anxious, younger, less likely to have a university degree and more likely to have smoked in pregnancy [48]. Implications of missing a disproportionate number of children exposed to high levels of anxiety may lead to an underestimation of the long-term effect of more severe perinatal anxiety.

### Mechanisms that mediate or moderate child emotional outcomes

There is a well-established association between prenatal and postnatal maternal distress, and consequently, prenatal exposures will co-vary with postnatal exposures [3]. Therefore, it needs to be determined whether maternal prenatal anxiety, which is presumed to affect the developing fetus through ‘fetal programming’, has a significant, unique effect on child outcomes above and beyond known associations, such as parenting behaviours [51, 52]. Anxiety and depression symptoms also often co-occur and the presence of co-morbidity is a marker of severity [53]. Inadequate measurement of or control for postnatal maternal anxiety or co-morbid postnatal depression could result in the misattribution of postnatally mediated mechanisms to prenatal biological ones and the majority of studies failed to adequately consider these potential mechanisms.

It is also difficult to separate the effects of maternal perinatal anxiety from the consequence of other factors that might contribute to child emotional problems that were not measured in many of the included studies. For example, the reviewed studies could not rule out potential genetic factors that might affect the observed association. Genetically informed studies involving children conceived through in vitro fertilisation who were not genetically related to their mothers provide strong evidence that the environment contributes to poor child mental health including anxiety risk [54, 55]. The impact of prenatal exposure to psychotropic medication also needs to be considered. In a recent paper from the Norwegian Mother and Child cohort study investigating the effect of prenatal exposure to selective serotonin reuptake inhibitors (SSRIs), the findings suggest that there was no substantial increased risk for externalizing, emotional, or social problems in preschool-aged children following prenatal SSRI exposure [56]. However, an association between prenatal psychotropic drug exposure and increased rates of depression diagnoses in older offspring has previously been documented [57], so this requires further study.

Only one study included endocrine (cortisol) measures to test for potential underlying mechanisms consistent with the fetal programming hypothesis [48]. In this study, maternal cortisol and psychosocial distress exerted independent effects on child mental health [49]. The other studies in this review did not examine physiological factors. Therefore, direct assessments of the physiological processes that may explain the observed associations and have been implicated by animal and human research (e.g., fetal programming) cannot be determined by this review. Similarly, only eight studies examined gender differences with very mixed results, so the impact of gender on the association between maternal perinatal anxiety and child emotional outcomes is inconclusive.

### Shared method variance

All studies had methodological issues related to shared method variance, which reduced their quality ratings. The use of self-report questionnaires meant that often mothers were reporting on their own levels of anxiety and also on their perceptions of their child’s behaviour. This could lead to mothers with elevated symptoms of anxiety and depression over- or misreporting their child’s emotions and mothers who do not experience anxiety not recognising symptoms in their children. Across the included studies, the effect of maternal anxiety on child emotional problems was most profound when mothers had reported on their child’s behaviour. However, only a minority of studies used multiple informants on the child outcome measure, for example, fathers [38, 41] or teachers [42, 43, and 44]. Murray et al. [40] and Prenoveau et al. [47] were the only studies to use observational measures of child outcomes. In addition to the effect of shared method variance, there is also the impact of context on understanding and interpreting children’s behaviours and emotional experience: for example, the parents knowing their child across multiple settings compared to teachers who in turn will be more able to view a child’s behaviour and emotional experience in comparison with peers [58, 59]. Nevertheless, it is likely that shared method variance partly explains the greater associations when mothers report on their own anxiety symptoms and their child’s emotional problems.

### Implications for future research

Future studies should incorporate multiple informants and employ a range of methods to measure children’s emotional problems (e.g., standardised clinical interviews, bio-markers of child emotional problems, and observational data). Biological measures would enable future studies to examine the association between both biological and psychological factors during gestation and the risk for adverse emotional outcomes in childhood. Similarly, the inclusion of postnatal risk factors such as quality of parenting should be incorporated as a potential mediator or moderator of the association between perinatal anxiety and children’s emotional problems. Future research should also examine the role of timing of exposure to prenatal anxiety (e.g., number of week gestation) as well as clinically significant levels of maternal anxiety as identified through diagnostic interviews.

### Implications for clinical practice

The finding that maternal anxiety during the perinatal period was associated with adverse emotional outcomes in children provides support for a preventative approach to infant developmental problems. Historically, much attention has been paid to postnatal depression, which has focused its approach on prevention and intervention beginning during the postnatal period. This review has demonstrated that anxiety experienced during the prenatal period has significant consequences for a child’s emotional development, highlighting the need for prenatal interventions for maternal mental health. It is possible that addressing maternal mental health, including anxiety in pregnancy, may in turn affect the mother’s relationship with her child and the overall family functioning, with widespread effects. These results were found in community-based samples, suggesting that mental health assessment and intervention are important components of routine perinatal care. This review provides health and economic-related arguments to support increased screening and access to specialist perinatal mental health services, with long-term implications for women’s mental health, child development, and well-being.

### Strengths and limitations of the current review

The findings of this review are the result of a thorough, systematic process reviewing a large number of articles. The inclusion of studies that measured anxiety symptoms alongside those that measured clinical diagnoses of anxiety is a strength of the review. The results highlight the importance of measurement of different forms of anxiety when considering the impact on child emotional problems. Despite the strengths of this review, some limitations exist. Only articles published in English were included, which means that relevant articles published in other languages may have been overlooked. It is also acknowledged that the use of a quality assessment tool involves a degree of subjectivity in the ratings process. Furthermore, inconsistent presentation of the results across the included studies rendered a meta-analysis difficult and beyond the scope of the current paper.

## Conclusion

There is some evidence that prenatal and postnatal anxiety exposure may lead to adverse emotional outcomes in children, but further evidence is needed. Expanding on the literature and improving the methodological rigour of such studies will enable a better understanding of the effects of maternal anxiety during the perinatal period on child emotional outcomes. Such research could lead to the improved identification of at-risk parents and children with appropriate opportunities for intervention and prevention.
